# Unraveling the molecular mechanism of novel leukemia mutations on NTRK2 (A203T & R458G) and NTRK3 (E176D & L449F) genes using molecular dynamics simulations approach

**DOI:** 10.12688/f1000research.131013.2

**Published:** 2024-06-18

**Authors:** Abeer M Al-Subaie, Balu Kamaraj, Fazil Ahmad, Khaldoon Alsamman

**Affiliations:** 1 Department of Clinical Laboratory Sciences, College of Applied Medical Sciences, Imam Abdulrahman Bin Faisal University, DAMMAM, Saudi Arabia; 2 Department of Dental Education, College of Dentistry, Imam Abdulrahman Bin Faisal University, DAMMAM, Saudi Arabia; 3 Department of Anesthesia Technology, College of Applied Medical Sciences in Jubail, , Imam Abdulrahman Bin Faisal University, Jubail, Saudi Arabia

**Keywords:** Leukemia; Mutations; Stability; Structural loss; Molecular mechanism; Modeling; Molecular dynamic simulations.

## Abstract

**Background**: NTRK1, NTRK2, and NTRK3 are members of the neurotrophic receptor tyrosine kinases (NTRK) family, which encode TrkA, TrkB, and TrkC receptors, respectively. Hematologic cancers are also linked to point mutations in the NTRK gene's kinase domain. Trk fusions are the most common genetic change associated with oncogenic activity in Trk-driven liquid tumors. Thus, point mutations in NTRK genes may also play a role in tumorigenesis. The structural and functional effect of mutations in Trk-B & Trk-C proteins remains unclear.

**Methods**: In this research, Homology (threading-based approach) modeling and the all-atom molecular dynamics simulations approaches are applied to examine the structural and functional behavior of native and mutant Trk-B and Trk-C proteins at the molecular level.

**Results:** The result of this study reveals how the mutations in Trk-B (A203T & R458G) and Trk-C (E176D & L449F) proteins lost their stability and native conformations. The Trk-B mutant A203T became more flexible than the native protein, whereas the R458G mutation became more rigid than the native conformation of the Trk-B protein. Also, the Trk-C mutations (E176D & L449F) become more rigid compared to the native structure.

**Conclusions:** This structural transition may interrupt the function of Trk-B and Trk-C proteins. Observing the impact of NTRK-2/3 gene alterations at the atomic level could aid in discovering a viable treatment for Trk-related leukemias.

## Introduction

NTRK1, NTRK2, and NTRK3 are members of the neurotrophic receptor tyrosine kinases (NTRKs) gene family, which code for the TrkA, TrkB, and TrkC receptors, respectively. ^1^ Gene fusions of NTRK genes correspond to the primary molecular changes with known carcinogenic and transformative abilities. ^2^ NTRK is detected in 1,500 to 5,000 children, teenagers, and adults with cancer yearly. ^1^ In-frame deletion of NTRK1 in acute myeloid leukemia has been described as a less frequent oncogenic pathway. ^1^^,^^3^ A recent study indicated that 5% of persons with diverse hematologic neoplasms, including acute myeloid leukemia, lymphoblastic leukemia, and myeloproliferative disorders, have NTRK2/3 point mutations. Regardless of where the tumor is in the body, neurotrophic tyrosine receptor kinase (NTRK) gene fusions are an actionable biomarker for cancer therapy and are present in over 25 cancer types. ^2^^,^^4^ However, the frequency of NTRK gene fusions differs among various tumor types. ^2^ A wide variety of solid tumor types, including breast, cholangiocarcinoma, colorectal, gynecological, neuroendocrine, non-small cell lung, salivary gland, pancreatic, sarcoma, and thyroid cancers, have been associated with NTRK fusion-positive tumors. ^1^^,^^4^

The NTRK2 gene, which codes for a protein called the TrkB receptor, with 822 amino acid residues, has 24 exons and is located on chromosome 9q22.17. ^5^ The total length of the TrkB receptor contains the following domains as follows: N-terminal signal sequence, cysteine-rich domain; leucine-rich domain, two immunoglobulins (Ig)-like domains that make up the BDNF-binding region, transmembrane domain, Src homology two domains containing (SHC)-binding motif, T.K. domain near the C terminus and a C-terminal PLCγ-docking site. ^5^^,^^6^ TrkC receptor is the transcription product of the NTRK3 gene and was discovered and characterized in 1991. ^7^^,^^8^ The NTRK3 gene is located on chromosome 15q25. ^7^^–^^9^ The total length of the TrkC receptor with 839 amino acid residues represented in the human's cerebral cortex, granular cell layer, and hippocampus. ^1^

Nine distinct point mutations in the NTRK2 or NTRK3 genes were reported in the recent study, many of which were outside the kinase domain. ^10^ Four of the nine distinct mutations can potentially cause cancer, and cells transformed by these mutations could be inhibited by Trk. ^10^ The mutation NTRK2 ^A203T^, located in the extracellular domain, was identified in a patient with primary myelofibrosis. ^10^ In addition, the mutation NTRK2 ^R458G^ is situated in the juxtamembrane domain with two individuals, one with chronic myeloid leukemia (CML) and the other with atypical CML. ^10^ The mutation NTRK3 ^E176D^ was detected in a patient with NPM1-mutated AML and located in the extracellular domain. The mutation NTRK3 ^L449F^ was observed in a patient with T-cell ALL and located in the transmembrane domain. ^10^ On the other hand, Trk fusions are the most frequent genetic alteration connected to carcinogenic activity in Trk-driven liquid tumors. Thus, point mutations in the NTRK genes may also contribute to the development of tumors.

Point mutations in NTRK genes may also contribute to the tumorigenic process, even though Trk fusions correspond to the essential genetic alterations that impart oncogenic activity in Trk-driven liquid tumors. However, this has not yet been thoroughly examined. However, their functional importance is yet unknown. In this study, the all-atom molecular simulations approach uses to observe the structural and functional behavior of native and mutant Trk-B (A203T & R458G) and Trk-C (E176D & L449F) proteins at the atomic level. Our findings will help scientists better understand the molecular causes of Trk-B and Trk-C protein mutations and pave the way for developing potentially tailored treatments for Trk-related leukemia patients.

## Methods

### Dataset

The four novel mutations of NTRK2 (A203T & R458G) and NTRK3 (E176D & L449F) genes were retrieved from a recent study. ^10^ In addition, the protein sequence of Trk-B (UNIPORT ID: Q16620) and Trk-C (UNIPROT ID: Q16288) were obtained from the UNIPROT database in FASTA format. ^11^ It helps to model the three-dimensional structure of native and mutant Trk-B and Trk-C proteins.

### Native and Mutant Trk-B and Trk-C proteins modeling

The NTRK2 and NTRK3 genes encoding proteins Trk-B and Trk-C do not have the PDB structures for the entire length. Hence, we modeled the Trk-B and Trk-C proteins from the amino acid sequences using the I-TASSER ^12^ program, which has been deemed an accurate and effective method. It is a threading-based method for predicting structures that could construct the protein's three-dimensional configurations. It produces a high quality modeled three-dimensional (3D) structures and biological processes of proteins from their amino acid sequences. It has also predicted the five 3-D model structures for the submitted amino acid sequences and selected the best model structure with the lowest energy. We inserted the point mutations to the predicted Trk-B and Trk-C model's 3D structures to further examine the effects of mutations on Trk-B and Trk-C proteins and run the energy minimization to generate the best mutant protein structures using the SWISS PdbViewer tool. Further, the quality of the projected modeled structures of the native and mutant Trk-B and Trk-C proteins was evaluated using the PROCHECK ^13^ and PROSA ^14^ programs.

### Molecular Dynamics simulations

The Molecular Dynamics simulations (MDS) were carried out using the GROMACS program. ^15^ We applied the default parameters from our earlier studies ^16^^–^^20^ for the MDS experiments, which are detailed below. The native and mutant Trk-B (A203T & R458G) and Trk-C (E176D & L449F) protein structures were used as MDS's starting point. The simulation used the CHARMM 36 ^21^ force field. The TIP3P water molecules were placed 10 Å from the box's edges to solvate the native and mutant Trk-B and Trk-C protein systems. Further, we used the genion tool to neutralize both the native and mutant Trk-B and Trk-C proteins. The energy minimization process used the steepest descent method to produce a stable protein conformation. ^22^ The electrostatic interactions were calculated using the particle mesh Ewald method. ^23^ The Berendsen coupling technique was used to regulate the temperature inside the box. ^24^ Further, two different equilibration procedures for the NVT (500 ps) and NPT (500 ps) were carried out. The water and non-water molecules are controlled during the equilibration using the Parrinello-Rahman barostat pressure coupling method ^25^ and LINCS ^26^ algorithms.

Finally, based on the convergence of native and mutant Trk-B and Trk-C protein system, the simulation was performed up to 50 nanoseconds (ns). Further, to inspect the structural behavior of Trk-B and Trk-C proteins upon mutations, we examined the root mean square deviation (RMSD), root mean square fluctuation (RMSF), radius of gyration (Rg), solvent accessible surface area (SASA) and the number of hydrogen bonds (H-bonds) analysis. To determine the covariance value of native and mutant Trk-B and Trk-C proteins, we used principal component analysis (PCA) ^27^^,^^28^ to assist our molecular dynamics (MD) analysis. PCA analysis is a technique that reduces data complexity and extracts concerted motions in simulations that are correlated and likely significant for biological function. During PCA analysis, a variance/covariance matrix is constructed from the trajectories after removing rotational and translational movements. This matrix is then diagonalized to identify a set of eigenvectors and eigenvalues. The eigenvalues represent the amplitude of the eigenvectors in multidimensional space, and the displacement of atoms along each eigenvector indicates the concerted motions of the protein in each direction. XMGRACE, ^29^ is a tool used to create the simulation plots.

## Results

### Prediction of native and mutant Trk-B and Trk-C protein's 3D structures

**Figure 1.**
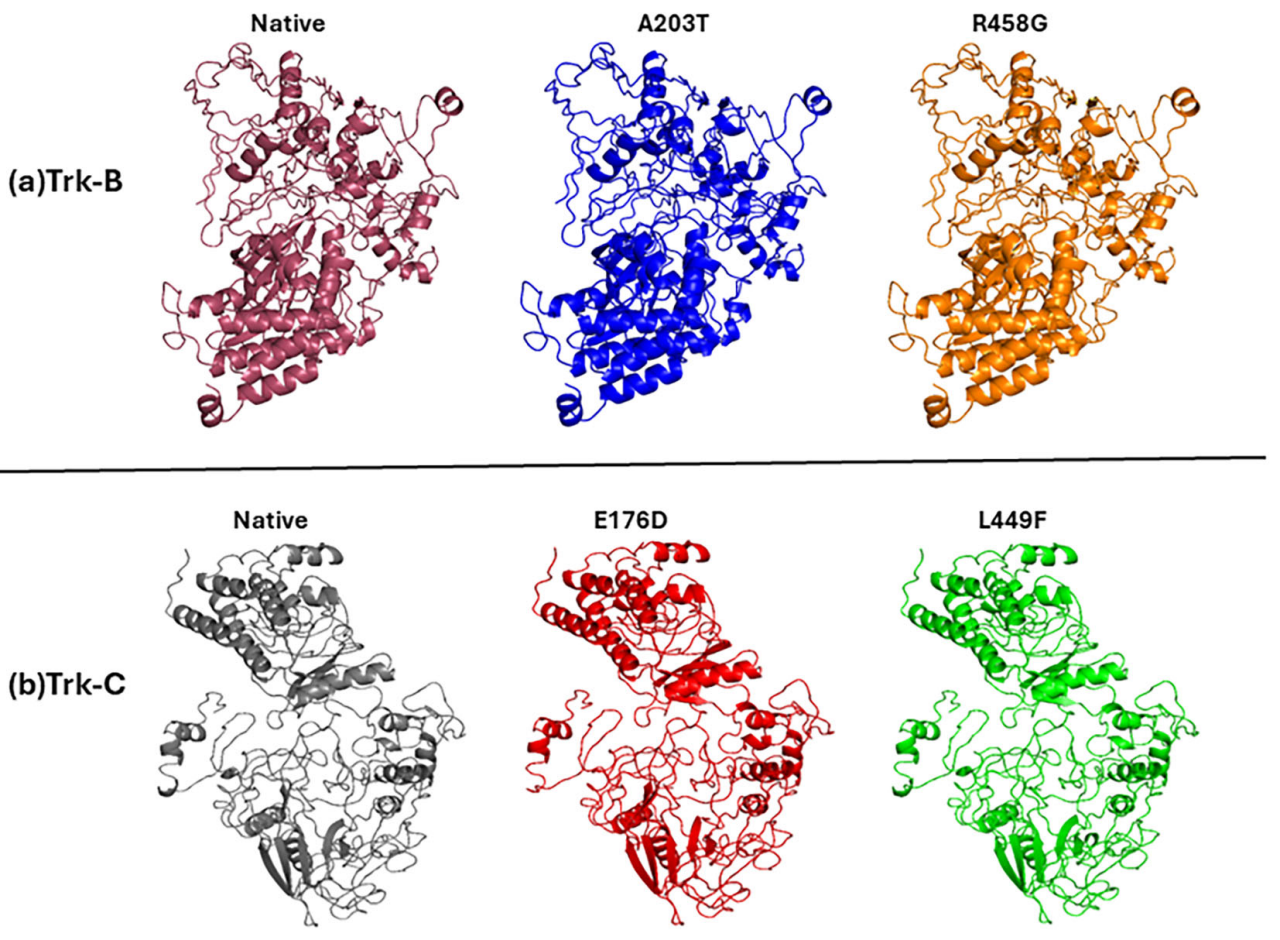
(a) Model structures of native and mutant (A203T & R458G) Trk-B proteins. (b) Model structures of native and mutant (E176D & L449F) Trk-C proteins.

To accurately predict the 3D conformation protein structures without missing residues, it is crucial to observe how Trk-B and Trk-C proteins alter their conformation in response to mutations. As a result, we rebuilt the 3D structures of the Trk-B and Trk-C proteins using the I-TASSER server. The algorithm used more than ten templates individually to model the Trk-B and Trk-C proteins. The topmost template (PDB ID: 5KMK_A) ^30^ showed 74% similarity and less than 36% sequence coverage to the Trk-B protein sequence. Similarly, the PDB ID: 4FL2_A ^31^ is a template showing 70% similarity and less than 41% sequence coverage to the Trk-C protein sequence. The best model structures of Trk-B and Trk-C proteins were obtained, based on the high confidence c-score, from I-TASSER. Furthermore, the mutant Trk-B (A203T & R458G) and Trk-C (E176D & L449F) structures build using the SWISS-PDB program. The accuracy of the predicted model structures of native and mutant Trk-B and Trk-C proteins was assessed by PROCHECK and PROSA programs. With a z-score value of -9.11 and a 98.8% favored and allowed region, the native Trk-B protein was examined. However, 98.1% of the favored and allowed regions were present in the mutant Trk-B (A203T & R458G) structures, and the z-score values ranged from -9.09 to -9.12. Similar results were obtained using native Trk-C protein, which had a 96.6% favored and allowed region and a z-score value of -6.54. At the same time, mutant Trk-C (E176D & L449F) structures displayed z-score values between -6.49 and -6.53 and favored and allowed regions with 95.2-95.4%, respectively. These results confirm the high degree of confidence in the predicted native and mutant modeled structures used for further study. The predicted model structures of native and mutant Trk-B and Trk-c are displayed in Figure 1a-b respectively.

### MD simulation of native and mutant Trk-B and Trk-C proteins

**Figure 2.**
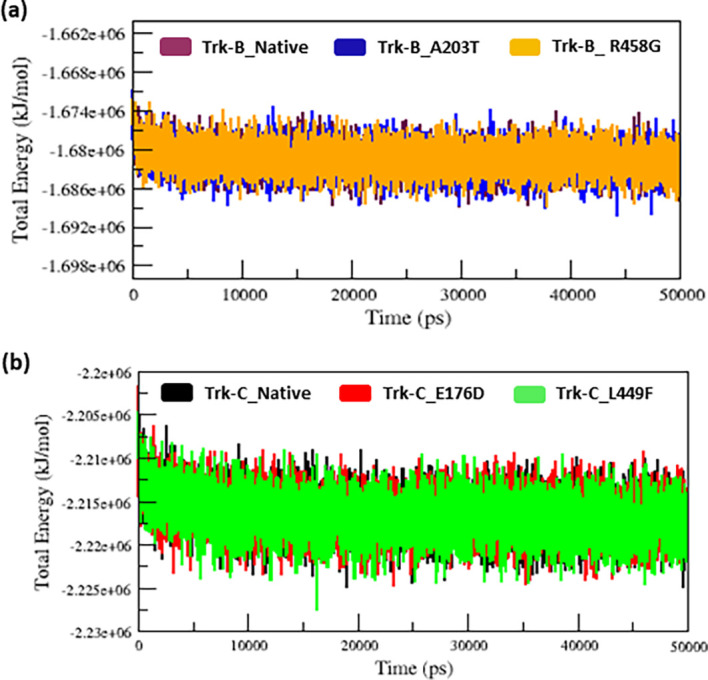
(a) The native and mutant (A203T & R458G) Trk-B total energy Vs. Time for 50 ns. (b) The native and mutant (E176D & L449F) Trk-C total energy Vs. Time for the period of 50 ns.

We used the MDS technique to examine the atom-level structural alterations in native and mutant Trk-B (A203T & R458G) and Trk-C (E176D & L449F) proteins. To evaluate the inconsistencies in structural and functional changes between the native and mutant Trk-B and Trk-C proteins, we investigated the RMSD, RMSF, Rg, SASA, H-bonds, and PCA analysis. In MD simulation, the total energy of native and mutant Trk-B and Trk-C proteins was measured from the beginning structures to examine the convergence of the protein system. As a result, the native and mutant Trk-B protein system ( Figure 2a) shows a similar way of deviation from the beginning to end of the simulation time. Similarly, the native and mutant Trk-C protein systems ( Figure 2b) exhibit a similar way of deviation from the beginning to the end of the simulation time.

**Figure 3.**
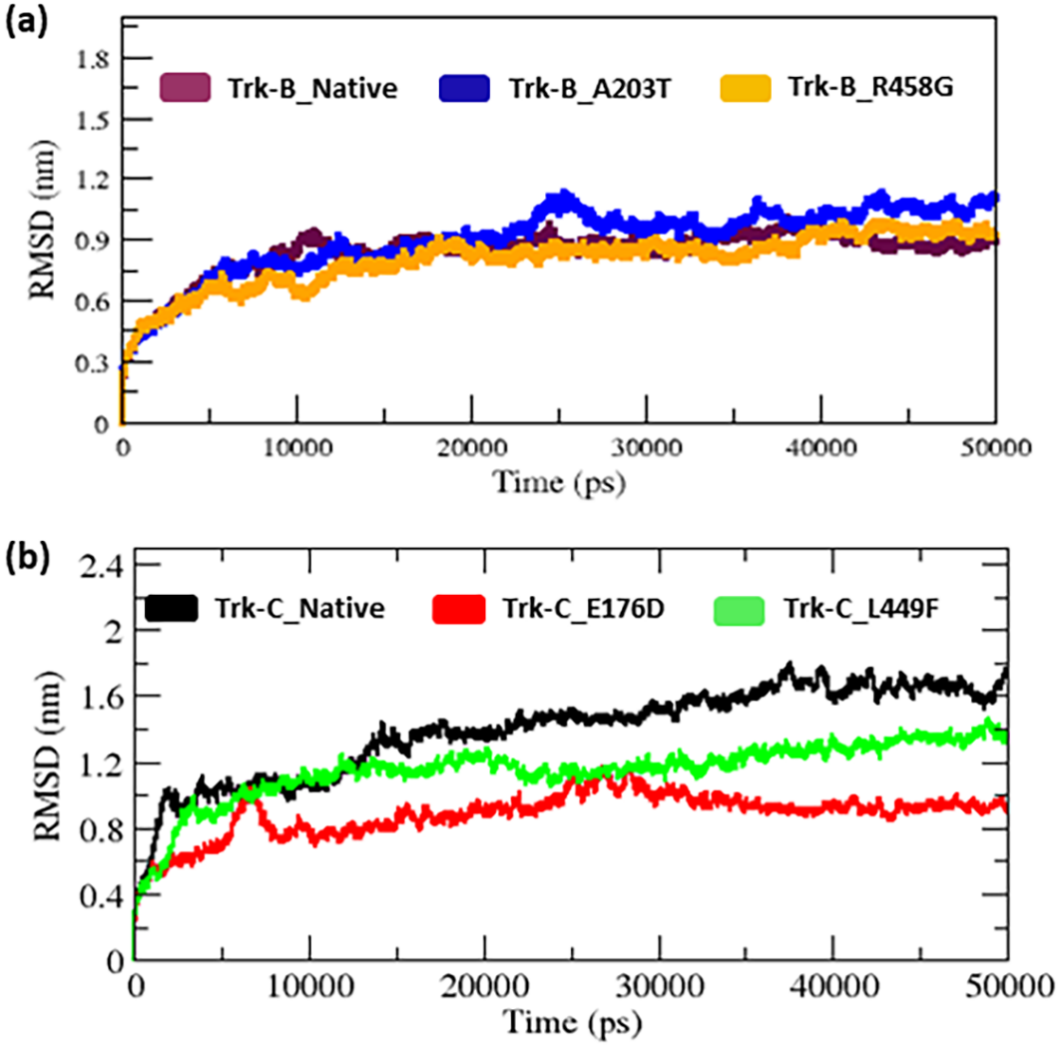
(a) The native and mutant (A203T & R458G) Trk-B backbone RMSD for the period of 50 ns. (b) The native and mutant (E176D & L449F) Trk-C backbone RMSD for the period of 50 ns.

Further, the RMSD for all Cα atoms from the initial structure was examined for the native and mutant Trk-B and Trk-C proteins ( Figure 3a). From the beginning to ~6 ns in the RMSD plot, the native and mutant Trk-B structures exhibit a similar pattern of variation, but from that, the A203T mutant Trk-B structure indicates a progressive increase in RMSD value in comparison to the native Trk-B structure until the end of the simulation time (50 ns) ( Figure 3a). At the same time, the R458G mutant Trk-B structures show a gradual decrease in RMSD compared to native Trk-B structures until the end of the simulation (50 ns).

**Figure 4.**
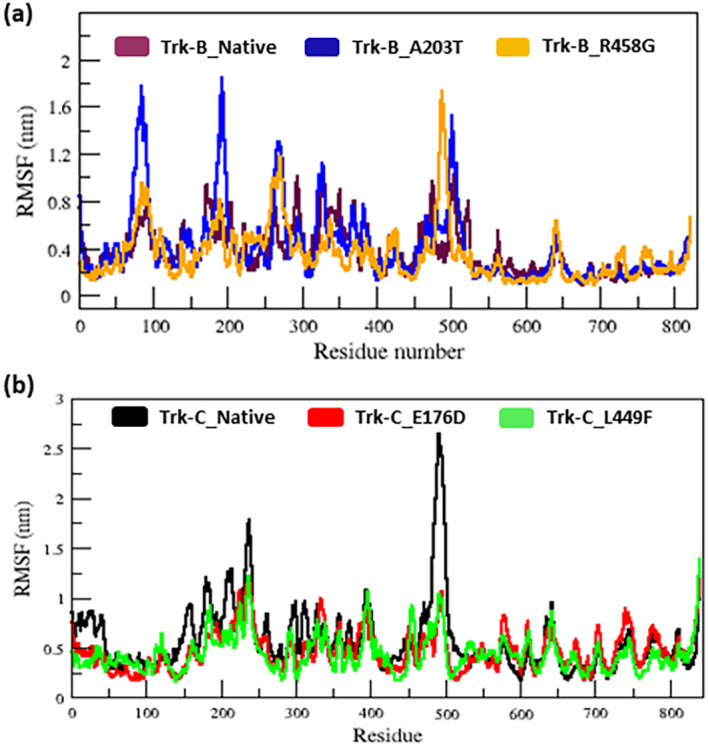
(a) The native and mutant (A203T & R458G) Trk-B protein residues in C-α RMSF simulation data for the period of 50 ns. (b) The native and mutant (E176D & L449F) Trk-C protein residues of C-α RMSF simulation data for the period of 50 ns.

Table 1 lists the average RMSD values for the native and mutant (A203T & R458G) Trk-B structures. Whereas native and mutant (E176D & L449F) Trk-C structures vary from the start to 50 ns simulation time ( Figure 3b). Both mutant (E176D & L449F) structures show a decrease in RMSD value compared to native Trk-C structures ( Figure 3b). Table 2 lists the average RMSD values for the native and mutant (E176D & L449F) Trk-C structures. The RMSF value of native and mutant structures of both Trk-B (A203T & R458G) & Trk-C (E176D & L449F) proteins show significant changes in the structures. ( Figure 4a-b). The A203T and R458G mutants showed a higher and lower degree of flexibility between the residues than the native Trk-B protein ( Figure 4a). Trk-C mutant structures (E176D & L449F) showed lower flexibility between the residues than the native protein ( Figure 4b). Table 1 & 2 lists the average RMSF values for native and mutant Trk-B (A203T & R458G) and Trk-C (E176D & L449F) proteins, respectively.

**Table 1.** Average RMSD, RMSF, Rg, SASA, H-bonds, and covariance value of native and mutant Trk-B.

Parameters	Native	A203T	R458G
RMSD	0.84 ± 0.12	0.90 ± 0.17	0.80 ± 0.13
RMSF	0.38 ± 0.19	0.41 ± 0.30	0.34 ± 0.23
R.G.	3.07 ± 0.05	3.11 ± 0.03	3.06 ± 0.02
SASA	516.43 ± 14.40	524.06 ± 13.77	511.94 ± 17.89
NH-bond(P-P)	399.22 ± 13.59	398.43 ± 12.94	399.84 ± 17.66
Co-variance	436.65	623.76	409.63

**Table 2.** Average RMSD, RMSF, Rg, SASA, H-bonds, and covariance value of native and mutant Trk-C.

Parameters	Native	E176D	L449F
RMSD	1.39 ± 0.28	0.88 ± 0.14	1.15 ± 0.19
RMSF	0.58 ± 0.34	0.47 ± 0.20	0.44 ± 0.18
R.G.	3.66 ± 0.11	3.61 ± 0.01	3.52 ± 0.07
SASA	545.06 ± 11.36	509.45 ± 7.63	532.99 ± 13.64
NH-bond(P-P)	384.11 ± 8.70	397.86 ± 11.59	408.49 ± 12.36
Co-variance	1124.33	649.56	562.33

**Figure 5.**
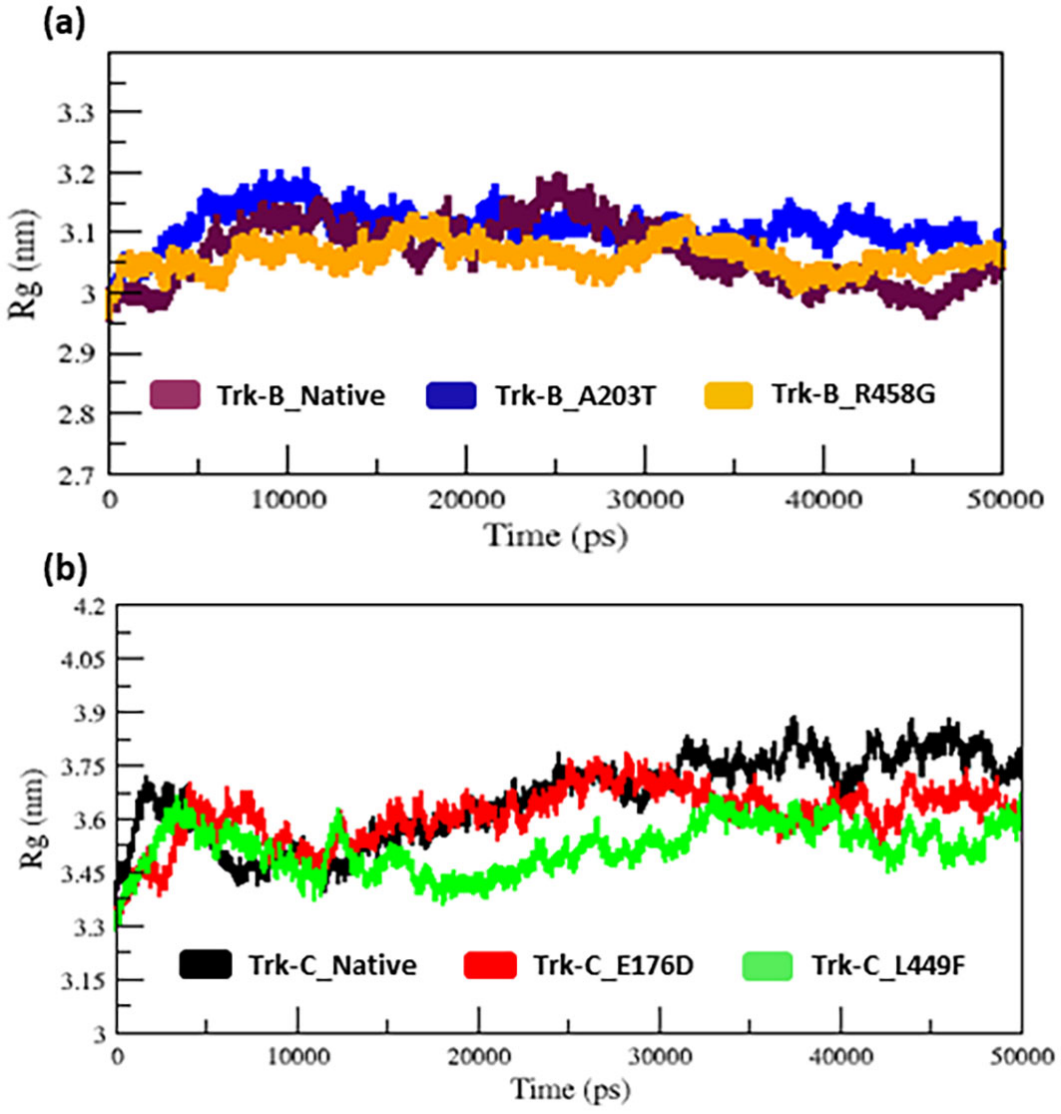
(a) The native and mutant (A203T & R458G) Trk-B protein compactness analysis by Rg for the period of 50 ns. (b) The native and mutant (E176D & L449F) Trk-C protein compactness analysis by Rg for the period of 50 ns.

**Figure 6.**
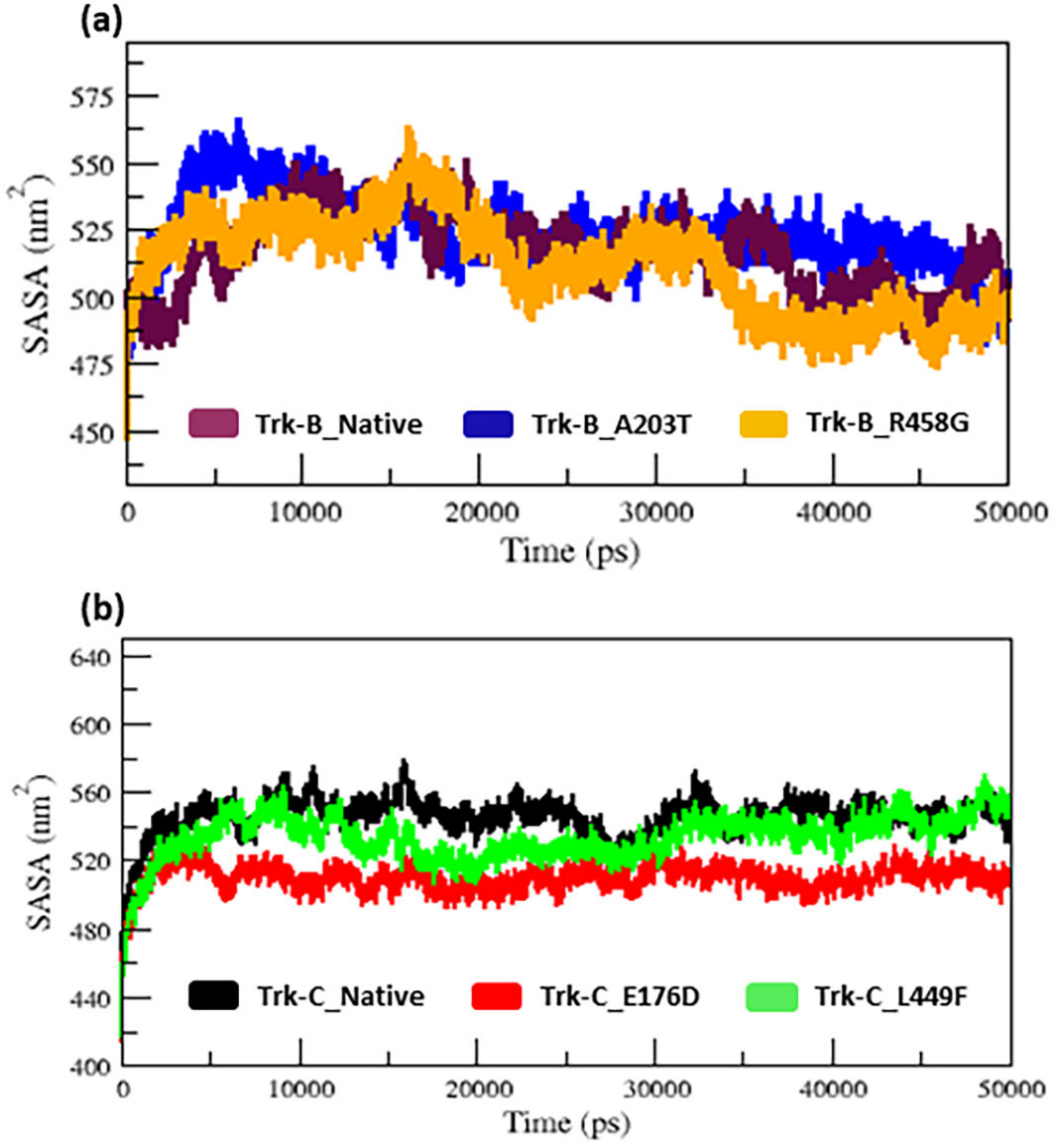
(a) The SASA analysis of native and mutant (A203T & R458G) Trk-B protein structures for the period of 50 ns. (b) The SASA analysis of native and mutant (E176D & L449F) Trk-C protein structures for the period of 50 ns.

The radius of gyration and SASA analysis provide compactness in the protein system. Figure 5a–b & 6a-b displays the Rg and SASA plot for C-alpha atoms in native and mutant Trk-B (A203T & R458G) and Trk-C (E176D & L449F) proteins overtime at 300 K. Tables 1 and 2 again list the average Rg and SASA values for the native and mutant Trk-B (A203T & R458G) and Trk-C (E176D & L449F) proteins, respectively. In Figures 5a & 6a, the A203T mutant structure shows similar Rg and SASA values from the beginning to ~1.5 ns, later which increases and leads to more Rg and SASA values than native Trk-B structure up to 50 ns simulation time. The R458G mutant structure starts with the same Rg value from the beginning to ~1 ns, after which it declines and displays less Rg value from ~1 ns to ~30 ns, and then it shows a similar way of Rg value up to the end of the simulation (50 ns) ( Figure 5a). Correspondingly, the R458G mutant structure offers an equal SASA value from the start of simulation to ~35 ns, later which decreases and displays less SASA value up to 50 ns simulation time ( Figure 6a).

**Figure 7.**
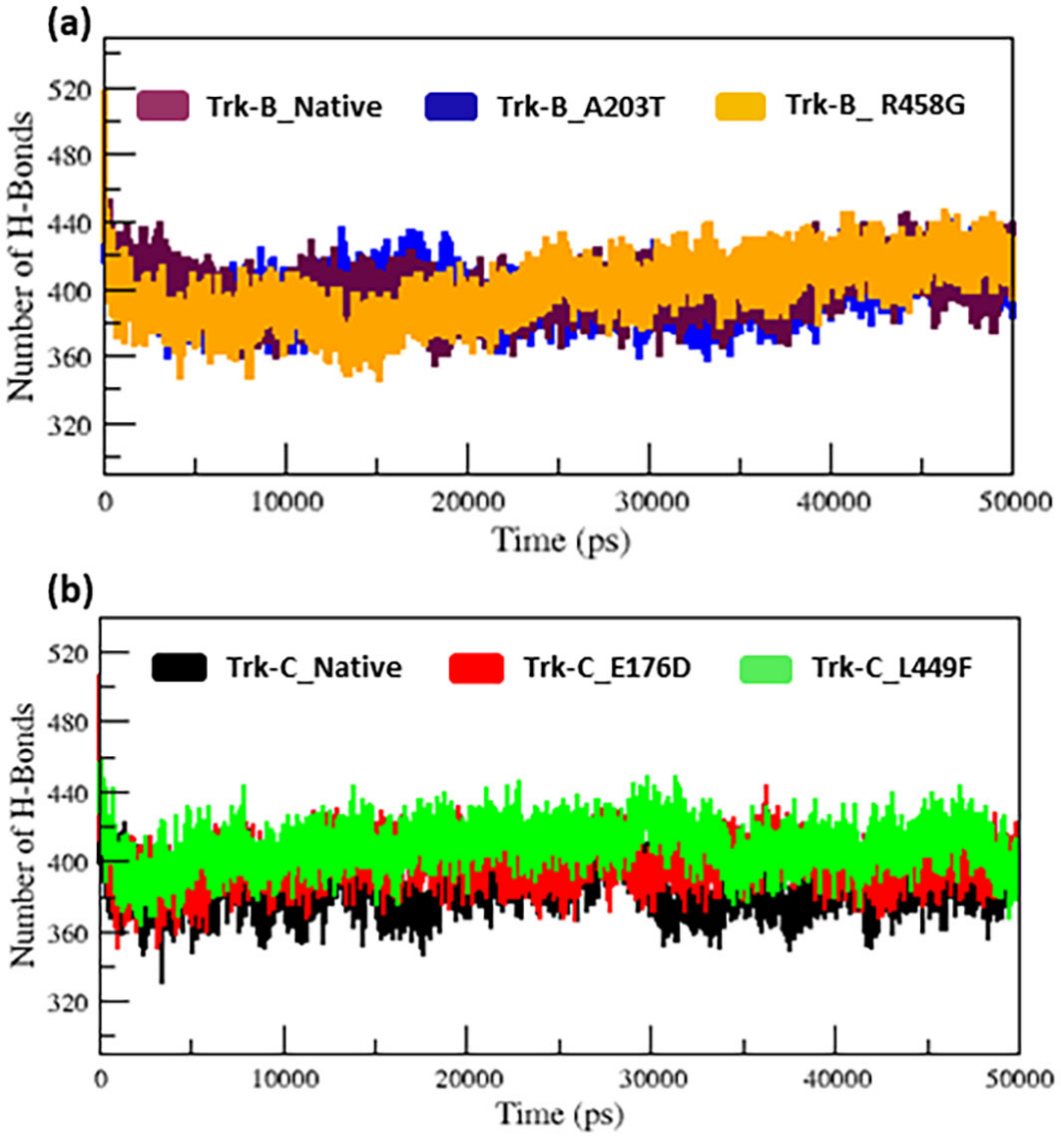
(a) The H-bond simulation data for the native and mutant (A203T & R458G) Trk-B protein structures for the period of 50 ns. (b) The H-bond simulation data for the native and mutant (E176D & L449F) Trk-C protein structures for the period of 50 ns.

In Figure 5b, from the start to ~32 ns, the E176D mutant shows a similar Rg value compared to the native Trk-C structure, but after that, the E176D mutant shows a decrease in the Rg value than the native structure up to the 50 ns simulation time. Likewise, the SASA value of the E176D mutant displays the same deviation to the native Trk-C structure from the beginning to ~3 ns, after which it decreases and shows less SASA value up to the end of the simulation ( Figure 6b). On the other hand, the L449F mutant exhibits the same Rg and SASA value as the native structure from 0 to ~14 ns after it decreases and offers less Rg value compared to the native Trk-C structure until 50 ns simulation time ( Figure 5b & 6b). The h-bond is necessary for protein folding, stability, and functionality. Therefore, we counted the number of H-bonds shown in Figure 7a-b to better understand the stability of native and mutant Trk-B and Trk-C proteins. Tables 1 and 2 display the average hydrogen bond values of the native and mutant structures of the Trk-B and Trk-C proteins.

Figure 7a indicates that the native Trk-B has slightly more H-bonds than the A203T mutant. The R458G mutant exhibits slightly more H-bonds than the native Trk-B protein. E178D and L449F mutants exhibit greater h-bonds in Figure 7b than native Trk-C protein ( Figure 7b).

**Figure 8.**
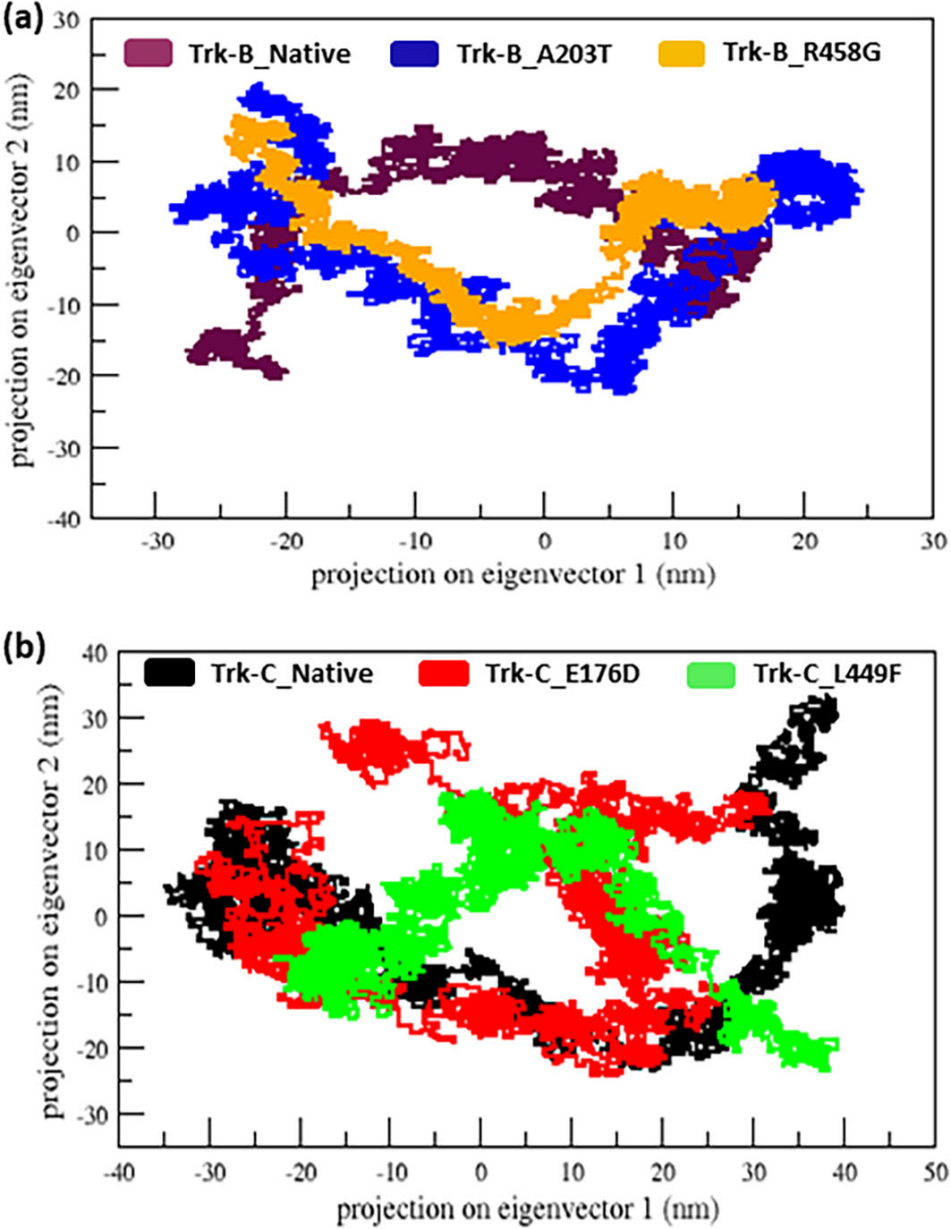
(a) Projection motion of the native and mutant (A203T & R458G) Trk-B protein structures for the period of 50 ns. (b) Projection motion of the native and mutant (E176D & L449F) Trk-C protein structures for the period of 50 ns.

**Figure 9.**
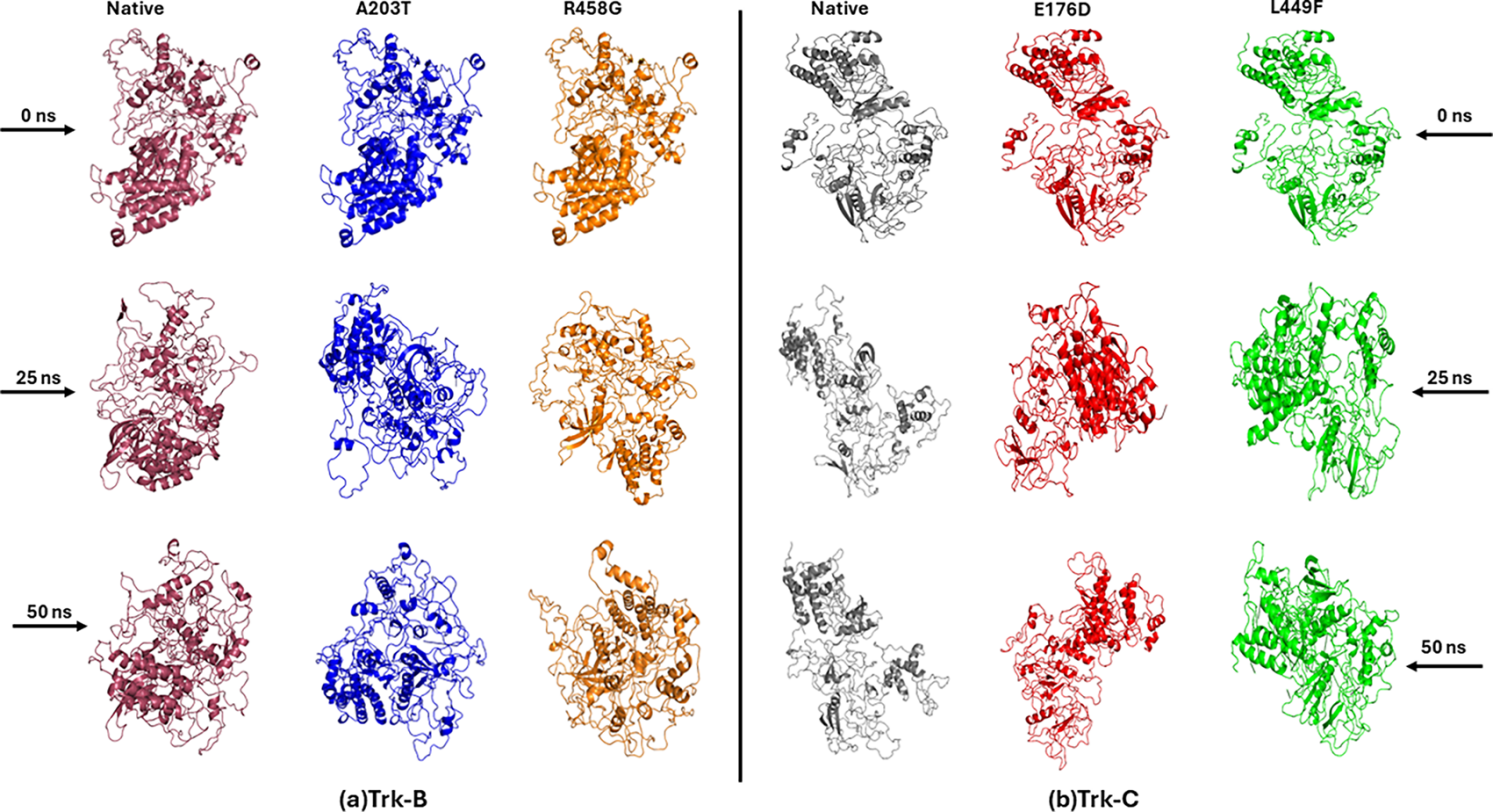
(a) Snapshots of native and mutant (A203T & R458G) Trk-B protein conformation at different simulation time scale (b) Snapshots of native and mutant (E176D & L449F) Trk-C protein conformation at different simulation time scale.

Further, we used the parameters based on our earlier studies ^32^^,^^33^ to perform the PCA analysis. ^27^^,^^28^ It is used to view the motion of Trk- B and Trk-C proteins upon mutations. In Figure 8a, the projection of the first two eigenvectors shows that the A203T mutant covers a more region phase space in both PC1 and PC2 plains compared to the native Trk-B protein. Whereas the R458G mutant covers a reduced region space in both PC1 and PC2 than the native Trk-B protein ( Figure 8a). Similarly, the E176D and L449F mutants span a reduced region phase in the PC1 and PC2 plain than the native Trk-C protein, as shown in Figure 8b. Furthermore, we displayed the native and mutant Trk-B and Trk-C protein structures at different timescale ( Figure 9a-b).

## Discussion

The 3-D conformation Trk-B and Trk-C proteins must be determined to investigate the structural behavior of novel mutations on the NTRK2 (A203T & R458G) and NTRK3 (E176D & L449F) genes. Then, the I-TASSER program is applied to model the Trk-B and Trk-C protein structures. After, the mutant Trk-B (A203T & R458G) and Trk-C (E176D & L449F) proteins are created using the SWISS PDB program. Finally, the PROSA & PROCHECK programs were applied to estimate the quality of native and mutant Trk-B and Trk-C proteins. To examine the structural significance of Trk-B and Trk-C proteins upon mutations, all the native and mutant Trk-B & Trk-C proteins were exposed to the timescale of 50 ns MD simulation. This research examined the structural changes in the Trk-B and Trk-C proteins resulting from mutations using the output trajectory from 0 to 50 ns. Between the native and mutant Trk-B and Trk-C proteins, we measured the total energy, RMSD, RMSF, Rg, SASA, and H-bonds. They are shown in Figures 2 to 7, respectively. Further, we assessed native and mutant Trk-B and Trk-C proteins using principal component analysis (PCA) ( Figure 8a-b). The average values for the native and mutant Trk-B and Trk-C protein's RMSD, RMSF, Rg, SASA, NH-bond, and covariance are depicted in Tables 1 and 2, respectively.

In MD simulation, the total energy of native and mutant Trk-B and Trk-C structures was measured from the beginning structures to examine the stability of the protein system. As a result, the native and mutant Trk-B proteins converged and showed a stable conformation ( Figure 2a-b) which was used for further analysis. It indicates that all the protein trajectories are stable and used for further investigations. However, in Figure 3a RMSD plot, the A203T mutant structure shows more deviation. Furthermore, the R458G mutant structure exhibited minor variation than the native Trk-B protein ( Figure 3a). At the same time, the Trk-C RMSD plot, both the mutant (E176D and L449F) systems show a minor deviation from than native TrK-3 protein ( Figure 3b). This result indicates that, due to mutations, both Trk-B and Trk-C proteins might undergo the structural transition, which affects the protein's function. Furthermore, the RMSF value of the native and mutant Trk-B and Trk-C proteins reveals significant structural alterations. ( Figure 4a-b). The A203T and R458G mutants showed a higher and lower degree of flexibility between the residues than the native Trk-B protein ( Figure 4a). It further illustrates that the mutant A203T lost their native conformation and became more flexible and the mutant R458G become rigid ( Figure 4b). On the other hand, Trk-C mutant structures (E176D & L449F) showed lower flexibility between the residues than native protein ( Figure 4b). It indicates that both the mutant structures of Trk-C protein lost their confirmation and became more rigid.

The radius of gyration and solvent accessibility of surface analysis provide compactness in the protein system. The Rg and SASA value of native and mutant structures of both Trk-B & Trk-C proteins are shown in Figure 5a-b & Figure 6a-b, respectively. The mutant A203T shows higher Rg and SASA values than native Trk-B ( Figure 5a & Figure 6a). The mutant R458G shows a lower Rg and SASA value than native Trk-B ( Figure 5a & Figure 6a). While Trk-C mutant proteins (E176D & L449F) show lower Rg and SASA values than native proteins ( Figure 5b & Figure 6b). The average hydrogen bond values of native and mutant structures of both Trk-B & Trk-C proteins are shown in Figure 7a-b. In Figure 7a, the A203T mutant shows a lower H-bond, and the mutant R458G shows a higher H-bond than the native Trk-B protein. In Figure 7b, both the mutant structures (E176D & L449F) show higher H-bonds than the native Trk-C protein. These NH-bond results are well associated with RMSD, RMSF, SASA, and Rg plots. Thus, the A203T mutant enlarges in Trk-B protein, and the R458G mutant is shrunken than the native Trk-B structure. At the same time, the Trk-C protein mutations are shrunken compared to the native structure.

Further, we performed PCA analysis to observe the motion of Trk-B and Trk-C proteins upon mutations. The Trk-B mutant A203T, show more movement and leads to flexible conformation. Whereas the R458G mutant shows less motion and leads to rigid confirmation ( Figure 8a). Both Trk-C mutations (E176D & L449F) show less movement and lead to rigid conformation ( Figure 8b). The structural changes of native and mutant Trk-B and Trk-C proteins at different simulation timescales (0 ns, 25 ns, & 50 ns) were observed and displayed in Figure 9a-b. It clearly thus, it confirms that mutation influences the structural behavior of the Trk-B and Trk-C proteins. Further, it could affect the function of NKRT2/3 genes and is responsible for causing leukemia and other hemopoietic malignancies.

## Conclusion

This research studied the structural and functional consequences of native and mutant Trk-B and Trk-C protein structures. Due to mutations, the Trk-B protein loses its stability. For example, the Trk-B mutant A203T is more flexible than the native protein, whereas the R458G mutation is more rigid than the native Trk-B conformation. In addition, compared to the native structure, the Trk-C mutations (E176D & L449F) become more rigid. As a result, the Trk-B and Trk-C protein functions may be affected due to structural change. This study will aid experimental laboratory scientists in better comprehending the molecular mechanism of novel NTRK2/3 gene mutations. Researchers may be able to use this information to establish a therapeutic target for NTRK2/3 gene-related leukemia.

## Data Availability

Figshare. Unraveling the molecular mechanism of novel leukemia mutations on NTRK2 (A203T & R458G) and NTRK3 (E176D & L449F) genes using molecular dynamics simulations approach. https://doi.org/10.6084/m9.figshare.22232218.v1. ^34^ This project contains the following underlying data:
• native_Trk-B.pdb (The modeled Trk-B protein structure). • A203T_Trk-B.pdb (The modeled mutant (A203T) Trk-B protein structure). • R458G_Trk-C.pdb (The modeled mutant (R458G) Trk-B protein structure). • Nat_Trk-C.pdb (The modeled Trk-C protein structure). • E176D_Trk-C.pdb (The modeled mutant (E176D) Trk-C protein structure). • L449F_Trk-C.pdb (The modeled mutant (L449F) Trk-C protein structure). • Trk_B-energy.xvg (The native and mutant (A203T & R458G) Trk-B total energy Vs. Time for 50 ns) • Trk_b-PCA.xvg (Projection motion of the native and mutant (A203T & R458G) Trk-B protein structures for the period of 50 ns). • Trk_B-PPhbond.xvg (The H-bond simulation data for the native and mutant (A203T & R458G) Trk-B protein structures for the period of 50 ns). • Trk_B-Rg.xvg (The native and mutant (A203T & R458G) Trk-B protein compactness analysis by Rg for the period of 50 ns.) • Trk_B-rmsd.xvg (The native and mutant (A203T & R458G) Trk-B backbone RMSD for the period of 50 ns). • Trk_b-rmsf.xvg (The native and mutant (A203T & R458G) Trk-B protein residues in C-α RMSF simulation data for the period of 50 ns). • Trk_b-SASA.xvg (The SASA analysis of native and mutant (A203T & R458G) Trk-B protein structures for the period of 50 ns). • Trk_C-2dproj_ev_1_2.xvg (Projection motion of the native and mutant (E176D & L449F) Trk-C protein structures for the period of 50 ns **).**
• Trk_C-energy.xvg (The native and mutant (E176D & L449F) Trk-C total energy Vs. Time for the period of 50 ns). • Trk_C-PPhbond.xvg (The H-bond simulation data for the native and mutant (E176D & L449F) Trk-C protein structures for the period of 50 ns). • Trk_C-Rg.xvg (The native and mutant (E176D & L449F) Trk-C protein compactness analysis by Rg for the period of 50 ns). • Trk_C-rmsd.xvg (The native and mutant (E176D & L449F) Trk-C backbone RMSD for the period of 50 ns). • Trk_C-rmsf.xvg (The native and mutant (E176D & L449F) Trk-C protein residues of C-α RMSF simulation data for the period of 50 ns). • Trk_C-SASA.xvg (The SASA analysis of native and mutant (E176D & L449F) Trk-C protein structures for the period of 50 ns). Data are available under the terms of the Creative Commons Attribution 4.0 International license (CC-BY 4.0).
