# Supplementary material for: Unraveling the molecular mechanism of novel leukemia mutations on NTRK2 (A203T & R458G) and NTRK3 (E176D & L449F) genes using molecular dynamics simulations approach
Source: F1000Res. 2024 Jun 18;12:345. Originally published 2023 Mar 28. [Version 2] doi: 10.12688/f1000research.131013.2 (PMC11307142; doi:10.12688/f1000research.131013.2)
Supplement: Peer review history

# Reviewer report: Reviewer response for version 2

**DOI:** 10.5256/f1000research.167790.r292453

**Published:** 2024-06-19

**Authors:** Gurudeeban Selvaraj

**Affiliations:** 1 Centre for Research in Molecular Modeling (CERMM), Department of Chemistry and Biochemistry, Concordia University, Montreal, Québec, Canada

The authors addressed all the comments raised by the reviewer. I accept the current form.

Is the work clearly and accurately presented and does it cite the current literature?

Yes

If applicable, is the statistical analysis and its interpretation appropriate?

Yes

Are all the source data underlying the results available to ensure full reproducibility?

Partly

Is the study design appropriate and is the work technically sound?

Yes

Are the conclusions drawn adequately supported by the results?

Yes

Are sufficient details of methods and analysis provided to allow replication by others?

Partly

Reviewer Expertise:

Computational Biology and Chemistry

I confirm that I have read this submission and believe that I have an appropriate level of expertise to confirm that it is of an acceptable scientific standard.

# Reviewer report: Reviewer response for version 2

**DOI:** 10.5256/f1000research.167790.r292452

**Published:** 2024-06-19

**Authors:** Rajasekaran R.

**Affiliations:** 1 Quantitative Biology Lab, Department of Integrative Biology, School of Bio Sciences and Technology, Vellore Institute of Technology (VIT, Deemed to be University), Vellore, Tamil Nadu, India

Authors addressed all the comments and now this manuscript can be indexed in its present form.

Is the work clearly and accurately presented and does it cite the current literature?

Yes

If applicable, is the statistical analysis and its interpretation appropriate?

Yes

Are all the source data underlying the results available to ensure full reproducibility?

Yes

Is the study design appropriate and is the work technically sound?

Yes

Are the conclusions drawn adequately supported by the results?

Yes

Are sufficient details of methods and analysis provided to allow replication by others?

Yes

Reviewer Expertise:

Computational Biology

I confirm that I have read this submission and believe that I have an appropriate level of expertise to confirm that it is of an acceptable scientific standard.

# Reviewer report: Reviewer response for version 1

**DOI:** 10.5256/f1000research.143816.r168093

**Published:** 2024-06-07

**Authors:** Rajasekaran R.

**Affiliations:** 1 Quantitative Biology Lab, Department of Integrative Biology, School of Bio Sciences and Technology, Vellore Institute of Technology (VIT, Deemed to be University), Vellore, Tamil Nadu, India

Abeer *et al.* focused on unravelling the molecular mechanism of novel leukaemia mutations on NTRK2 (A203T & R458G) and NTRK3 (E176D & L449F) genes using a molecular dynamics simulations approach. Initially, the authors have used the various bioinformatics tools to screen the mutations which causing tooth agenesis.

 Further they implemented modeling and an MDS approach to elucidate the structural changes of the Trk-B and Trk-C proteins upon mutations. This study helps to understand the molecular mechanism of Trk-B and Trk-C proteins upon mutations at the atomic level. It could help the researchers to establish personalized drugs for NTRK2/3 gene-related leukaemia. Overall, the paper is well-written and explained. So, I recommend the article to be indexed.

**Minor comments:**

 Ø  Cite the reference for the force field (CHARMM 36) used in the method section of the MD simulation.

 Ø  Display the modeled native and mutant Trk-B and Trk-C protein structures in the article. 

Is the work clearly and accurately presented and does it cite the current literature?

Yes

If applicable, is the statistical analysis and its interpretation appropriate?

Yes

Are all the source data underlying the results available to ensure full reproducibility?

Yes

Is the study design appropriate and is the work technically sound?

Yes

Are the conclusions drawn adequately supported by the results?

Yes

Are sufficient details of methods and analysis provided to allow replication by others?

Yes

Reviewer Expertise:

Computational Biology

I confirm that I have read this submission and believe that I have an appropriate level of expertise to confirm that it is of an acceptable scientific standard.

# Response

**Published:** 2024-06-10

**Authors:** Balu Kamaraj

**Affiliations:** Imam abdulrahman bin faisal university, Saudi Arabia

We sincerely appreciate all your valuable comments and suggestions, which helped us in improving the quality of the manuscript.

 The responses to the minor comments of the reviewer are addressed below.

**Comment 1:** Cite the reference for the force field (CHARMM 36) used in the method section of the MD simulation.

**Response:** As per your valuable suggestion we have cited and listed the reference related to CHARMM 36 in the revised manuscript version. 

**Comment 2:** Display the modeled native and mutant Trk-B and Trk-C protein structures in the article.

**Response:** We agreed to your valuable suggestion. We have displayed the modeled structure of native and mutant Trk-B and Trk-C proteins in Figure 1 in the revised manuscript version.

# Reviewer report: Reviewer response for version 1

**DOI:** 10.5256/f1000research.143816.r168095

**Published:** 2024-06-06

**Authors:** Gurudeeban Selvaraj

**Affiliations:** 1 Centre for Research in Molecular Modeling (CERMM), Department of Chemistry and Biochemistry, Concordia University, Montreal, Québec, Canada

The study titled "Unraveling the molecular mechanism of novel leukemia mutations on NTRK2 (A203T & R458G) and NTRK3 (E176D & L449F) genes using molecular dynamics simulations approach" by Alsubaie *et al.* is a fascinating piece of research. The authors provide insights into the molecular mechanism of Trk-B and Trk-C proteins upon mutations using a molecular dynamics simulation approach. They also identify the effects of these mutations on the protein's structural conformation and its functional loss. Overall, the study presents exciting findings to the scientific audience. However, there are a few changes that need to be made for indexing purposes, as well as some areas that require further clarification:

1. The study's aim is missing from the abstract, and the method's tools and parameters for computational studies (modeling and MD simulation) are also absent. Additionally, modeling and structural quality assessments are not included in the results.
2. Please explain the reason for choosing the 5KMK_A and 4FL2_A protein structures as templates for modeling the TRK-B and Trk-C proteins. Also, provide predicted structures in the manuscript.
3. It would be helpful to visualize the native and mutant Trk-B protein structures at different time scales and discuss them in the manuscript.
4. It would also be beneficial to visualize the native and mutant Trk-C protein structures at different time scales and discuss them in the manuscript.
5. The authors may describe in detail the principal component analysis and XMGRACE analysis methods.
6. There are a number of typos (ex. higher region phase space) and grammar checks are required throughout the manuscript.
7. Better to use H-bonds instead of NH-bonds for the number of hydrogen bonds

Is the work clearly and accurately presented and does it cite the current literature?

Yes

If applicable, is the statistical analysis and its interpretation appropriate?

Yes

Are all the source data underlying the results available to ensure full reproducibility?

Partly

Is the study design appropriate and is the work technically sound?

Yes

Are the conclusions drawn adequately supported by the results?

Yes

Are sufficient details of methods and analysis provided to allow replication by others?

Partly

Reviewer Expertise:

Computational Biology & Chemistry

I confirm that I have read this submission and believe that I have an appropriate level of expertise to confirm that it is of an acceptable scientific standard, however I have significant reservations, as outlined above.

# Response

**Published:** 2024-06-10

**Authors:** Balu Kamaraj

**Affiliations:** Imam abdulrahman bin faisal university, Saudi Arabia

We thank the reviewer for sharing their valuable time and providing critical comments which is indeed very useful for improving the manuscript.

 The responses to the questions of the reviewer are included below.

**Comment 1:** The study's aim is missing from the abstract, and the method's tools and parameters for computational studies (modeling and MD simulation) are also absent. Additionally, modeling and structural quality assessments are not included in the results.

**Response:** Thanks for your valuable suggestion. We have mentioned the study aims in the abstract methods section and modeling and structural quality assessment are discussed in the abstract of the revised manuscript.

**Comment 2:** Please explain the reason for choosing the 5KMK_A and 4FL2_A protein structures as templates for modeling the TRK-B and Trk-C proteins. Also, provide predicted structures in the manuscript.

**Response**: In this study, we have used a threading-based approach (I-TASSER program) to model the Trk_B and Trk-C proteins. I-TASSER searches the template for the given Trk-B and Trk-C protein sequences individually against the protein data bank.  Based on the highest sequence similarity and coverage it predicts the templates individually to model the Trk-B and Trk-C proteins.  As a result, PDB ID: 5KMK_A showed 74% similarity and less than 36% sequence coverage to the Trk-B protein sequence and was considered as a template for modeling the Trk-B protein. Similarly, the PDB ID: 4FL2_A shows 70% similarity and less than 41% sequence coverage to the Trk-C protein sequence and is considered a template to model the Trk_B protein. Also, we have shown the modeled native and mutant Trk-B and Trk-C proteins in the revised manuscript as Figure 1.

**Comment 3**: It would be helpful to visualize the native and mutant Trk-B protein structures at different time scales and discuss them in the manuscript.

**Response**. We agree with your valuable suggestion. We have displayed the native and mutant Trk-B protein structures at different time scales as Figure 9a and discussed in the revised manuscript.

**Comment 4**: It would also be beneficial to visualize the native and mutant Trk-C protein structures at different time scales and discuss them in the manuscript.

**Response**: We agreed to your valuable suggestion. We have displayed the native and mutant Trk-C protein structures at different time scales as Figure 9b and discussed in the revised manuscript.

**Comment 5**: The authors may describe in detail the principal component analysis and XMGRACE analysis methods.

**Response**. Thank you for the suggestion. We have discussed the PCA and XMGRACE analysis in the revised manuscript.

**Comment 6**: There are a number of typos (ex. higher region phase space) and grammar checks are required throughout the manuscript.

**Response**: We have corrected the typos in the revised version.

**Comment 7**: Better to use H-bonds instead of NH-bonds for the number of hydrogen bonds

**Response**: As per your valuable suggestion, we have used H-bonds instead of NH-bonds in the revised manuscript.

# Reviewer report: Reviewer response for version 1

**DOI:** 10.5256/f1000research.143816.r168092

**Published:** 2024-06-04

**Authors:** Dicson Sheeja Malar

**Affiliations:** 1 Department of Clinical Chemistry, Faculty of Allied Health Sciences, Chulalongkorn University, Bangkok, Bangkok, Thailand

The study entitled 'Unraveling the molecular mechanism of novel leukemia mutations on NTRK2 (A203T & R458G) and NTRK3 (E176D & L449F) genes using a molecular dynamics simulations approach' was an original article that is well-organized and valuable. It observed the structural consequence of leukemia mutations on NTRK2 and NTRK3 genes.

 Methods included in this study are appropriate. First, the authors used a threading-based modeling technique to predict the 3-D conformational native and mutant Trk-B and Trk-C proteins. Hence it used multiple templates to predict the protein structure from sequences. Further, the authors used GROMACS tool to perform the all-atom simulation to observe the structural changes on Trk-B and Trk-C proteins.

 The results and discussion section explained well how the geometry of Trk-B and Trk-C protein structures changes due to mutations. In addition, the article gives new insights into NTRK2 and NTRK3 gene mutations related to leukemia. This information will help to develop potential drug targets in the future.

 Overall, the paper is well-framed and organized. Therefore, I approve the current form of this article for indexing.

Is the work clearly and accurately presented and does it cite the current literature?

Yes

If applicable, is the statistical analysis and its interpretation appropriate?

Not applicable

Are all the source data underlying the results available to ensure full reproducibility?

Yes

Is the study design appropriate and is the work technically sound?

Yes

Are the conclusions drawn adequately supported by the results?

Yes

Are sufficient details of methods and analysis provided to allow replication by others?

Yes

Reviewer Expertise:

Molecular Biology, Neurodegeneative diseases and Bioinformatics

I confirm that I have read this submission and believe that I have an appropriate level of expertise to confirm that it is of an acceptable scientific standard.

# Response

**Published:** 2024-06-10

**Authors:** Balu Kamaraj

**Affiliations:** Imam abdulrahman bin faisal university, Saudi Arabia

We’re grateful and appreciate you taking the time to share your feedback with us.
